# A novel preparation for histological analyses of intraventricular macrophages in the embryonic brain

**DOI:** 10.1111/dgd.12935

**Published:** 2024-06-19

**Authors:** Futoshi Murayama, Hisa Asai, Arya Kirone Patra, Hiroaki Wake, Takaki Miyata, Yuki Hattori

**Affiliations:** ^1^ Department of Anatomy and Cell Biology Nagoya University Graduate School of Medicine Nagoya Japan; ^2^ Department of Anatomy and Molecular Biology Nagoya University Graduate School of Medicine Nagoya Japan; ^3^ Department of Physiological Sciences, Graduate School for Advanced Studies SOKENDAI Hayama Japan; ^4^ Division of Multicellular Circuit Dynamics, National Institute for Physiological Sciences National Institute of Natural Sciences Okazaki Japan

**Keywords:** brain, cerebrum, cortex, macrophage, microglia

## Abstract

Microglia colonize the brain starting on embryonic day (E) 9.5 in mice, and their population increases with development. We have previously demonstrated that some microglia are derived from intraventricular macrophages, which frequently infiltrate the pallium at E12.5. To address how the infiltration of intraventricular macrophages is spatiotemporally regulated, histological analyses detecting how these cells associate with the surrounding cells at the site of infiltration into the pallial surface are essential. Using two‐photon microscopy‐based in vivo imaging, we demonstrated that most intraventricular macrophages adhere to the ventricular surface. This is a useful tool for imaging intraventricular macrophages maintaining their original position, but this method cannot be used for observing deeper brain regions. Meanwhile, we found that conventional cryosection‐based and naked pallial slice‐based observation resulted in unexpected detachment from the ventricular surface of intraventricular macrophages and their mislocation, suggesting that previous histological analyses might have failed to determine their physiological number and location in the ventricular space. To address this, we sought to establish a methodological preparation that enables us to delineate the structure and cellular interactions when intraventricular macrophages infiltrate the pallium. Here, we report that brain slices pretreated with agarose‐embedding maintained adequate density and proper positioning of intraventricular macrophages on the ventricular surface. This method also enabled us to perform the immunostaining. We believe that this is helpful for conducting histological analyses to elucidate the mechanisms underlying intraventricular macrophage infiltration into the pallium and their cellular properties, leading to further understanding of the process of microglial colonization into the developing brain.

## Graphical abstract

We found that conventional cryosection‐based and naked pallial slice‐based histological analyses resulted in unexpected detachment of intraventricular macrophages, which contribute to the seeding of some microglial population, from the ventricular surface and their mislocation in the ventricular space. Here, we report that a methodological preparation by pretreating brain slices with agarose‐embedding enabled us to delineate the structure of intraventricular macrophages and their interactions with the surrounding brain cells.

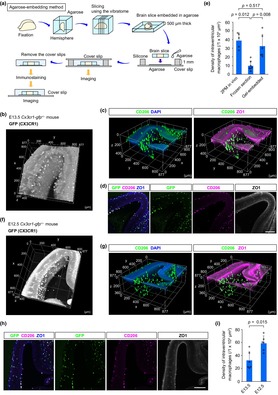

## 1 INTRODUCTION

Microglia, immune cells in the central nervous system (CNS), play multiple roles throughout life. In the adult brain, microglia regulate neuronal circuits by organizing synaptic formation and maintaining environmental homeostasis by removing cellular debris and apoptotic cells (Li & Barres, 2018; Paolicelli et al., 2011; Parkhurst et al., 2013; Wake et al., 2009). During development, these cells modulate neurogenesis (Arnò et al., 2014; Cunningham et al., 2013; Hattori & Miyata, 2018), the positioning of interneurons (Fujita et al., 2020; Squarzoni et al., 2014), and brain morphology (Lawrence et al., 2024). In mice, microglia originate from erythromyeloid progenitors in the yolk sac around embryonic day (E) 7.5–E8.5 and start to colonize the brain around E9.5 (Ginhoux et al., 2010; Prinz et al., 2019; Stremmel et al., 2018). However, it remains unclear which route they use to enter the brain. In the brain, there are two types of macrophages, microglia and CNS‐associated macrophages (CAMs), which are located at the interface of the vascular system and the meninges, ventricles, and choroid plexus (Prinz et al., 2021). Both originate from the erythromyeloid progenitors, but the timing of their fate is controversial (Dalmau Gasull et al., 2024). A previous study provided a model in which the fate of the yolk sac was determined based on the identification of two distinct cell types in yolk sac progenitors, which were characterized by the expression level of CD206, a specific marker for CAMs (Utz et al., 2020). On the other hand, another study using fate mapping showed that CD206^+^ yolk sac progenitors still have the ability to differentiate into both CAMs and microglia (Masuda et al., 2022), indicating that microglia do not stem only from CD206^−^ progenitors but are also supplied from early‐committed CD206^+^ progenitors. Furthermore, we recently reported that some microglia are derived from intraventricular macrophages, which easily infiltrate the pallium at E12.5, suggesting that microglia are composed of at least two cell populations that have different colonization routes (Hattori et al., 2023). Of note, the infiltration of intraventricular macrophages into the pallium is tightly regulated in a spatiotemporal manner: they preferentially infiltrate the pallium at E12.5, but they hardly enter the structure later than E13.5 in mice. It remains unclear how the infiltration of intraventricular macrophages is spatiotemporally regulated to form a microglial cell population in the pallium, so further studies are needed to assess the cellular and molecular mechanisms underlying the seeding process of microglial progenitors into the developing brain. We previously reported that intraventricular macrophages attach to the ventricular surface of the pallium, but we have encountered the problem that most of these cells are lost in conventional histological analyses. Hence, we sought to develop new histological approaches or to apply a combination of methods to determine how intraventricular macrophages interact with other brain cells when they infiltrate the pallium during a specific period.

## 2 MATERIALS AND METHODS

### 2.1 Experimental animals

*Cx3cr1‐gfp* mice (stock no. 005582, RRID: IMSR_JAX:005582) were purchased from Jackson Laboratories (Jung et al., 2000). ICR mice were purchased from Japan SLC. All mice were housed under specific pathogen‐free conditions at Nagoya University. The animal experiments were conducted according to the Japanese Act on Welfare and Management of Animals, Guidelines for Proper Conduct of Animal Experiments (published by the Science Council of Japan), and the Fundamental Guidelines for Proper Conduct of Animal Experiment and Related Activities in Academic Research Institutions (published by the Ministry of Education, Culture, Sports, Science, and Technology, Japan). All protocols for animal experiments were approved by the Institutional Animal Care and Use Committee of Nagoya University (No. 29006). To obtain *Cx3cr1‐gfp* ^+/−^ embryos, male *Cx3cr1‐gfp* homo mice were mated with female ICR mice. The day when the vaginal plug was detected was considered E0.5. Both male and female embryos were used, and similar results were obtained.

### 2.2 In vivo observation using two‐photon microscopy

**FIGURE 1:**
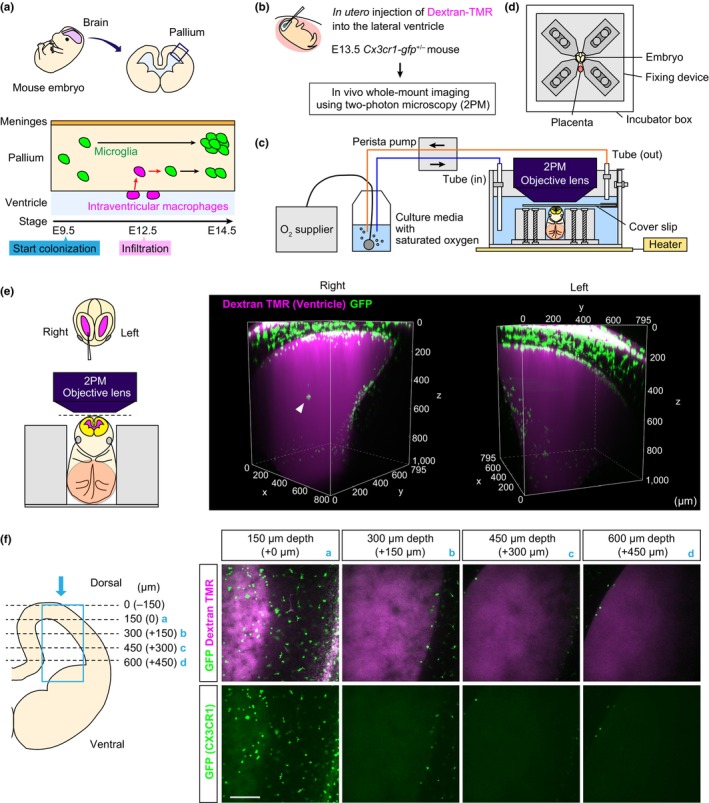
Whole‐embryo imaging using 2PM revealed a large number of intraventricular macrophages on the ventricular surface. (a) Schema depicting our current model of microglial colonization routes in the embryonic mouse brain. (b) Experimental procedure for in vivo scanning of CX3CR1^+^ cells in the brains of *Cx3cr1‐gfp* ^+/−^ embryos. E13.5 *Cx3cr1‐gfp* ^+/−^ mice were subjected to in utero intraventricular injection of dextran‐TMR to visualize the ventricular space. (c) Full setup of the in vivo ex utero imaging system for mouse embryos. The head of an E13.5 *Cx3cr1‐gfp* ^+/−^ mouse was scanned using 2PM. The embryo continued to live during the observation period by supplying sufficient oxygen with heat. (d) Image showing the top view of the fixing device, in which the embryo was set by four jigs in the center. (e) Three‐dimensional reconstructed whole‐embryo 2PM images covering an approximately 1‐mm depth from the dorsal part of the brain showing the attachment of CX3CR1^+^ cells to the ventricular surface of the pallium of the E13.5 *Cx3cr1‐gfp* ^+/−^ mouse. The right hemisphere was subjected to glass capillary injection with dextran‐TMR. (f) Horizontal 2PM scan of the left hemisphere of an E13.5 *Cx3cr1‐gfp* ^+/−^ mouse. Images were captured at 0, 150, 300, and 450 μm depths from the dorsal ventricular surface. Scale bar, 100 μm.

E13.5 *Cx3cr1‐gfp* ^*+/−*^ mice, which were injected with dextran‐tetramethylrhodamine (TMR) (Invitrogen, Cat# D1868) in the right lateral ventricle, were generated from the uterus of the mother. The embryo maintained the connection to the placenta to obtain an oxygen supply from the umbilical cord during the procedure (Figure 1b–d). Without oxygen, the embryo will die. The embryo with the placenta was immediately immersed in Dulbecco's modified Eagle's medium/F12 (fetal bovine serum‐free) culture medium (Sigma‐Aldrich, Cat# D2906) saturated with oxygen and was fixed in the device (Hattori Sada Ironworks Co., Ltd.) inside the incubator box. During the observation, the medium was continuously replaced with oxygen‐saturated medium via circulation between the incubator box and an attached bottle, which was bubbled with 40% O_2_ and 5% CO_2_, as previously reported (Hattori et al., 2023). Throughout the preparation and imaging processes, the embryo and incubator box were kept at 37°C with a heating plate that was set at the bottom to maintain body temperature. This method enabled us to perform continuous time‐lapse imaging for at least 3 h without any issues.

The brain hemispheres were scanned by two‐photon microscopy (2PM) based on a C2 plus (Nikon, Tokyo, Japan) with a Ti:sapphire laser (Coherence, Santa Clara, CA, USA) tuned to 950 nm and a 16× objective water immersion lens (N.A. 0.8; Nikon). The laser intensity was 3.0–15 mW (Figure 1e, f; Videos S1 and S2). The step size for each Z slice was 3 μm. The scanning was driven by a Galvano scanner.

### 2.3 Immunofluorescence

**FIGURE 2:**
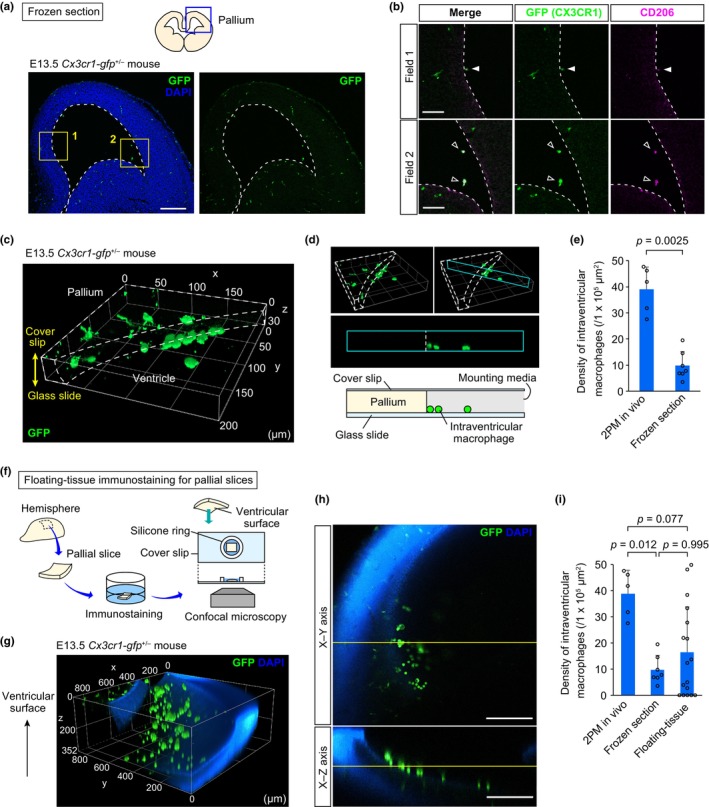
Difficulties in maintaining the density and position of intraventricular macrophages in conventional histological analyses. (a) Immunostaining for GFP in the cerebral wall of E13.5 *Cx3cr1‐gfp* ^+/−^ mice. Yellow squares indicate the magnified fields shown in (b). Broken line, ventricular surface contour. (b) Magnified images of the squared regions in (a) showing the positions of intraventricular macrophages (CX3CR1^+^ CD206^+^). Filled triangle, intraventricular macrophages attached to the pallial surface. Open triangles, those detached from the surface. (c) Three‐dimensional (3D) image of a 30‐μm‐thick frozen brain section from an E13.5 *Cx3cr1‐gfp* ^+/−^ mouse immunostained with an anti‐GFP antibody showing the accumulation of CX3CR1^+^ cells on glass slides (bottom panel). (d) Pictures (top) indicating the location of the cross‐sectional plane (cyan at the top right). A cross‐sectional view of the slice (middle). The cartoon (bottom) depicts the accumulation of intraventricular macrophages on glass slides, mimicking the middle picture. (e) Graph showing the density of intraventricular macrophages on the ventricular surface (two‐sided Mann–Whitney *U* test; six fields from two mice, seven fields from three mice [left to right]). (f) Schema showing the procedure of floating‐tissue‐based immunostaining for pallial slices. (g) 3D image of a whole pallial slice from an E13.5 *Cx3cr1‐gfp* ^+/−^ mouse, which was immunostained with an anti‐GFP antibody. The results revealed the nonhomogeneous distribution of CX3CR1^+^ cells on the ventricular surface. The ventricular surface faced the top. (h) The X‐Y axis (top) and X‐Z axis (bottom) images of the mouse pallial slice. (i) Graph showing the density of intraventricular macrophages on the ventricular surface (two‐sided Steel–Dwass test; five fields from two mice, seven fields from three mice, and 17 fields from four mice [left to right]). Scale bar, 200 μm (a, h), 50 μm (b).

The brains were fixed in 4% paraformaldehyde (PFA) for 1.5 h, immersed in 20% sucrose for 2–3 h, embedded in OCT compound (Sakura Finetek Japan Co., Ltd.), and then frozen. The brains were cut into 30‐μm thick sections on a cryostat. Sections were treated with the following primary antibodies overnight at 4°C: goat anti‐CD206 polyclonal antibody (pAb) (1:300, R&D Systems, Cat# AF2535, RRID: AB_2063012); rat anti‐GFP monoclonal antibody (mAb) (1:1000, Nacalai Tesque, Cat# GF090R, RRID: AB_2314545); and rabbit anti‐Iba1 pAb (1:1000, FUJIFILM Wako Pure Chemical Corp., Cat# 019–19741, RRID: AB_839504). After being rinsed, the sections were incubated with secondary antibodies conjugated to Alexa Fluor 488, 546, or 647 (1:1000, Invitrogen, Cat# A10036 [RRID: AB_2534012], Cat# A11056 [RRID: AB_2534103], Cat# A21206 [RRID: AB_2535792], Cat# A21208 [RRID: AB_2535794], Cat# A32787 [RRID: AB_2762830]) and then stained with DAPI (Sigma‐Aldrich, Cat# D9542). After being stained, the sections were mounted with mounting solution and sealed with coverslips. Slides were imaged by confocal microscopy with a TiEA1R (Nikon) and an AXR (Nikon) (Figure 2a–e). In all immunostaining experiments, the cell density of intraventricular macrophages was analyzed by counting the number of cells in the area covering the dorsolateral cerebral wall in the hemisphere.

### 2.4 Floating whole‐mount staining of brain slices

E13.5 *Cx3cr1‐gfp* ^+/−^ brains were fixed in 4% PFA overnight at 4°C. The next day, the pallial wall was sliced into square pieces using surgical scalpels. The excised pallial slices were rinsed with phosphate‐buffered saline containing 0.01% Triton X and then treated with a rat anti‐GFP mAb (1:1000, Nacalai Tesque, Cat# GF090R) overnight at 4°C. After the rinsing steps, the sections were treated with secondary antibodies conjugated to Alexa Fluor 488 (1:1000, Invitrogen, Cat# A21208) together with DAPI (Sigma‐Aldrich, Cat# D9542). After being stained, the slices were mounted with water inside a silicone ring on a coverslip and immediately scanned by confocal microscopy (AXR, Nikon) (Figure 2f–i).

### 2.5 Agarose‐embedding of brain slices

**FIGURE 3:**
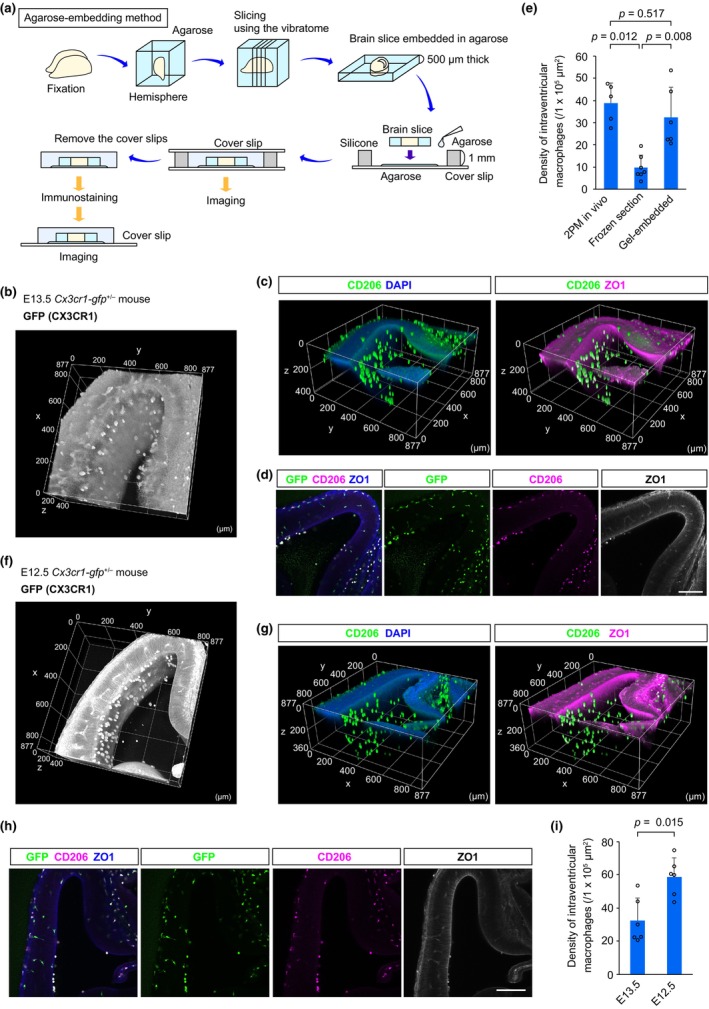
Agarose‐embedding preparation to stabilize intraventricular macrophages on the ventricular surface. (a) Image showing the procedure of agarose‐embedding preparation for brain slices. (b) Three‐dimensional (3D) image of agarose‐embedded brain slices from an E13.5 *Cx3cr1‐gfp* ^+/−^ mouse. Detection of GFP fluorescence revealed the massive presence of CX3CR1^+^ cells on the ventricular surface. (c) 3D images of agarose‐embedded E13.5 *Cx3cr1‐gfp* ^+/−^ pallial slices immunostained for CD206 (pseudo color green), ZO1 (magenta), and DAPI (blue; nucleus). (d) Two‐dimensional (2D) images of agarose‐embedded E13.5 *Cx3cr1‐gfp* ^+/−^ pallial slices immunostained for CX3CR1 (GFP; green), CD206 (magenta), and ZO1 (blue in merged picture, or white in each color indication). (e) Graph showing the density of intraventricular macrophages on the ventricular surface (two‐sided Steel–Dwass test; five fields from two mice, seven fields from three mice, and six fields from three mice [left to right]). (f) 3D image of agarose‐embedded slices from an E12.5 *Cx3cr1‐gfp* ^+/−^ mouse. (g) 3D images of agarose‐embedded E12.5 *Cx3cr1‐gfp* ^+/−^ pallial slices immunostained for CD206 (pseudo color green), ZO1 (magenta), and DAPI (blue; nucleus). (h) 2D images of immunostaining for CX3CR1 (GFP; green), CD206 (magenta), and ZO1 (blue in merged picture, or white in each color indication). (i) Graph showing the density of intraventricular macrophages on the ventricular surface (two‐sided Mann–Whitney *U* test; six fields from three mice). Scale bar, 200 μm (d, h).

The whole embryos were fixed in 4% PFA overnight at 4°C. The next day, the brain hemisphere embedded in 1% agarose gel was sliced with a vibratome at a thickness of 500 μm. The brain slice with agarose was placed on a coverslip on which four pillars (1‐mm thick) were set at the corners. The slices were mounted by adding further 1% agarose gel and covered with another coverslip. The coverslips were removed after the agarose gel had solidified, and then the agarose‐embedded sections were subjected to immunostaining in a 35‐mm culture dish. The samples were treated with goat anti‐CD206 pAb (1:300, R&D Systems, Cat# AF2535, RRID: AB_2063012), rat anti‐GFP mAb (1:1000, Nacalai Tesque, Cat# GF090R), and mouse anti‐ZO‐1 mAb (1:500, Thermo Fisher Scientific, Cat# 33‐9100, RRID: AB_87181) overnight at 4°C. After the rinsing steps, the samples were treated with secondary antibodies conjugated to Alexa Fluor 488, 546, and 647 (1:1000, Invitrogen, Cat# A11056 [RRID: AB_2534103], Cat# A21208 [RRID: AB_2535794], Cat# A32787 [RRID: AB_162542]) together with DAPI (Sigma‐Aldrich, Cat# D9542). After being stained, agarose‐embedded slices were placed on a coverslip and immediately scanned by confocal microscopy (AXR, Nikon) (Figure 3).

### 2.6 Statistical analysis

Quantitative data are presented as the mean values ± S.D.s of representative experiments. Statistical differences between groups were analyzed using R software by the Mann–Whitney *U* test for two‐group comparisons or the Steel–Dwass test for multiple comparisons. All the statistical tests were two‐tailed, and *p* < 0.05 was considered to indicate statistical significance. The *p* value is shown in each graph (n.s., not significant). Individual values are plotted as circles in the bar graphs. The number of samples examined in each analysis is shown in the corresponding figure legend. No randomization was used, and no samples were excluded from the analysis. No statistical methods were used to predetermine the sample size because of experimental limitations.

## 3 RESULTS AND DISCUSSION

We previously found that intraventricular macrophages, which frequently infiltrate the pallium at E12.5, serve as a source of some microglia in mice (Figure 1a). This highlights the need for more detailed studies combined with histological analyses of intraventricular macrophages to evaluate how these cells associate with the surrounding cells along the pallial surface while seeking when and where to enter the pallium. We previously established in vivo ex utero whole‐mount brain imaging for entire embryos without any surgical dissection using 2PM, which is especially useful for early embryonic stages, such as in E12.5 and E13.5 mice (Hattori et al., 2023). To examine where intraventricular macrophages are located inside the ventricular space, the E13.5 *Cx3cr1‐gfp* ^+/−^ mouse embryo, in which both microglia and macrophages are labeled with GFP (Jung et al., 2000), was imaged using this in vivo ex utero imaging system. The embryo contained in the mother's uterus was given dextran‐TMR in advance into the right lateral ventricle to visualize the ventricular space and then transferred to a culture box filled with culture medium containing sufficient oxygen to maintain embryo survival (Figure 1b–d). Scanning of the embryonic brain using 2PM demonstrated that almost all the intraventricular macrophages adhered to the ventricular surface of the pallium, did not float in the cerebrospinal fluid, in the left hemisphere, and were not damaged by glass capillary injection (Figure 1e; Video S1). In contrast, in the right hemisphere, which was subjected to the injection of dextran‐TMR, CX3CR1‐GFP^+^ cells were occasionally present as a clump within the ventricular lumen. These observations indicate that intraventricular macrophages exist along the ventricular surface under physiological conditions but are easily detached from the ventricular surface by surgical treatment.

Next, we evaluated how deep we were able to observe intraventricular macrophages in the brain using a 2PM‐based in vivo ex utero imaging system. By collecting Z‐axis sequential images of the left hemisphere of an E13.5 *Cx3cr1‐gfp* ^+/−^ mouse, we found that the GFP fluorescence of the cells was easily detected at a depth of 300 μm from the dorsal ventricular surface (at a depth of 450 μm from the brain surface) (Figure 1f; Video S2). However, detection of GFP fluorescence became more difficult at depths greater than 450 μm from the dorsal ventricular surface (at a depth of 600 μm from the top). These results suggest that 2PM‐based in vivo imaging, although very useful for capturing the physiological location of intraventricular macrophages, has limitations when visualizing the fine structure of cells far from the surface.

To investigate how intraventricular macrophages are associated with the surrounding cells along the ventricular surface, thin sample preparation is required to conduct a detailed structural analysis. However, immunostaining of frozen sections, a widely used histological technique, showed that most intraventricular macrophages were unexpectedly detached from the ventricular surface and/or moved from their original location. Immunostaining of frozen sections of E13.5 *Cx3cr1‐gfp* ^+/−^ mice showed that some intraventricular macrophages seemed to maintain their attachment to the ventricular surface, but some of them were located inside the ventricular lumen, apart from the ventricular surface (Figure 2a,b). Importantly, we recognized another serious problem: not only were the intraventricular macrophages partially dislodged, but they also tended to fall and accumulate on the glass even though they were still at the same X‐Y position (Figure 2c,d; Video S3). Moreover, comparison with 2PM‐based in vivo imaging revealed that the density of intraventricular macrophages attached to the pallial surface was markedly lower in frozen sections (Figure 2e). These results raised the possibility that intraventricular macrophages easily change their location from the physiological state during cryosectioning and/or subsequent immunostaining.

To avoid the aggregation or incorrect location of intraventricular macrophages through frozen‐section‐based histological analyses, we performed whole mount immunostaining using E13.5 *Cx3cr1‐gfp* ^+/−^ on free‐floating pallial slices, which were excised from the dorsolateral pallial wall (Figure 2f). The excised pallial slices were immediately fixed and subjected to immunostaining for GFP. Notably, we found that the density of intraventricular macrophages varied widely between the regions on the pallial slice, that is, these cells maintained their density in some regions corresponding to the density in 2PM‐based in vivo imaging, whereas other regions were almost empty (Figure 2g–i; Video S4), indicating less abundance and an uneven/heterogeneous distribution of intraventricular macrophages on the pallial ventricular surface in the floating‐tissue whole‐mount immunostaining method. Taken together, our findings showed that immunostaining procedures using conventional cryosection‐based and floating‐tissue‐based methods do not represent the actual position of intraventricular macrophages and simultaneously demonstrated the necessity of finding another strategy or improvements to the present methods to solve this problem.

To address the difficulty in collecting histological information on intraventricular macrophages through conventional frozen‐section‐based and floating‐tissue‐based methods, we sought to develop an improved method by modifying the floating‐tissue‐based method (Figure 3a). The brains of E13.5 *Cx3cr1‐gfp* ^+/−^ mice were first embedded in agarose and then sliced into 500‐μm‐thick slices using a vibratome. The slices were further embedded in agarose gel by gently dropping agarose on the tissue, followed by sealing with a coverslip. After the agarose had solidified, the coverslips on the sample were removed, and the sample was immediately scanned by confocal microscopy or subsequently subjected to immunostaining. We found that a massive number of intraventricular macrophages remained on the ventricular surface upon immediate scanning for GFP fluorescence (Figure 3b; Video S5). Importantly, even after immunostaining, the density of intraventricular macrophages, which were positive for CD206 (a marker for CAMs), was highly maintained on the ventricular surface (ZO‐1‐positive region) and was almost comparable to that obtained by 2PM‐based in vivo scanning. The positions of intraventricular macrophages were also successfully maintained in the slices that were pretreated with agarose (Figure 3c–e; Video S6). Hence, this method enabled us to compare the density of intraventricular macrophages between developmental stages. Indeed, we found that the density of intraventricular macrophages, which attach to the ventricular surface, at E12.5, was much greater than that at E13.5 (Figure 3f–i; Videos S7 and S8 **)**, suggesting that the high number of intraventricular macrophages might be one of the reasons why they can infiltrate the pallium at E12.5 (Hattori et al., 2023). Taken together, floating‐tissue‐based immunostaining combined with agarose‐embedding preparation successfully prevented the detachment and mislocation of intraventricular macrophages. This method is a useful histological approach for assessing intraventricular macrophage infiltration and contributes to the understanding of the detailed mechanisms that regulate microglial seeding in the developing brain.

Intraventricular macrophages exist in the ventricular space containing the cerebrospinal fluid within the brain and are inevitably exposed to the external environment once the tissues are excised from the brain. As these cells are not protected by the solid structure, they are easily detached from the pallial surface when triggered by any surgical simulation or treatment. We assume that cellular loss during conventional histological analyses and immunostaining methods might be attributed to the direct exposure of the cells to the many rinses in buffer solutions and clearing before the embedding compound is used. In addition, these cells could accidentally detach from the parenchymal surface when placing the frozen section onto the glass slide or before, that is, during fixation or sucrose cryoprotection. Here, we report that the key preparative step for histological analyses was to embed pallial slices in agarose before immunostaining. This step enabled us to avoid mechanical stimulation when processing frozen sections and rinsing, thus overcoming the limitations of conventional histological analyses. We observed that the physiological density and position of intraventricular macrophages subjected to histological analyses at this step were consistent with those of intraventricular macrophages, which was confirmed by the 2PM‐based in vivo imaging system. These findings may further our understanding of microglial development and colonization in the developing brain.

The reason for the preferential infiltration of intraventricular macrophages at E12.5 is currently unknown. As mentioned above, this might be because their density at E12.5 is greater than that at other developmental stages. Alternatively, extrinsic environmental factors, such as neural progenitors, which actively move their cell bodies by interkinetic migration (Miyata et al., 2014; Reiner et al., 2012), and blood vessels, which extend their thin processes toward the ventricular surface (Di Marco et al., 2020; Komabayashi‐Suzuki et al., 2019), might regulate their infiltration. The surrounding cells and/or structures might provide the scaffold or space for macrophage infiltration. Alternatively, intraventricular macrophages might acquire the ability to enter the pallium at E12.5. This method we reported would be helpful for evaluating such extrinsic and intrinsic factors through the collection of detailed information about tissue‐ and cellular‐level properties by maintaining the original position.

## AUTHOR CONTRIBUTIONS

F.M. and Y.H. designed the study. F.M. performed most of the experiments and data analysis. H.A. and A.K.P. assisted with data collection for immunohistochemical analyses. H.W. supported the 2PM observations. Y.H. wrote the paper. F.M., H.A., A.K.P., H.W., and T.M. reviewed the manuscript. Funding was acquired by H.W., T.M., and Y.H. All authors approved the final version of the manuscript and agreed to be accountable for all aspects of the work.

## CONFLICT OF INTEREST STATEMENT

The authors declare no competing interests.

## Supporting information


**Video S1.** In vivo imaging showed that intraventricular macrophages are attached to the ventricular surface. The right and left hemispheres of *Cx3cr1‐gfp* ^+/−^ E13.5 mouse, which was intraventricularly injected with dextran‐TMR in advance, scanned at 2PM (Figure 1e). The movie shows 3D reconstructed images covering a depth of approximately 1 mm from the dorsal brain surface to the ventral part.

**Video S2.** X‐Y sequential images of in vivo images of E13.5 *Cx3cr1‐gfp* ^+/−^ mice. Horizontal (X‐Y) images were obtained by 2PM scanning of the left hemisphere of an E13.5 *Cx3cr1‐gfp* ^*+/−*^ mouse, which was intraventricularly injected with dextran‐TMR (magenta) in advance (Figure 1f). The movie covers all the Z‐axis positions from the dorsal to ventral region (2.5 μm pitch). Scale bar, 100 μm.

**Video S3.** X‐Y sequential images of immunostained frozen sections. The X‐Y images show the position of CX3CR1^+^ cells in frozen sections of E13.5 *Cx3cr1‐gfp* ^+/−^ mice immunostained with an anti‐GFP antibody, indicating that the cells dropped onto glass slides (0.5 μm pitch) (Figure 2c).

**Video S4.** X‐Y sequential images of whole‐mount immunostained pallial slices. Horizontal (X‐Y) images of E13.5 *Cx3cr1‐gfp* ^+/−^ mouse pallial sections, immunostained with an anti‐GFP antibody, were obtained by confocal microscopy, which indicated that intraventricular macrophages were heterogeneously distributed on the ventricular surface (Figure 2h).

**Video S5.** 3D image of an E13.5 *Cx3cr1‐gfp* ^+/−^ mouse pallial slice after agarose‐embedding. The movie shows the 3D image of the E13.5 *Cx3cr1‐gfp* ^+/−^ pallial slice, which was prepared using the agarose‐embedding method, for detecting the GFP signal. This revealed that intraventricular macrophages maintained their number and position on the ventricular surface (Figure 3b).

**Video S6.** X‐Y sequential images of an immunostained E13.5 mouse pallial slice after agarose‐embedding. The movie shows dual signals for CD206 (pseudo color green) and DAPI (blue) in the pallial slices of immunostained E13.5 mice treated with agarose‐embedded preparation glass (5 μm pitch) (Figure 3c).

**Video S7.** 3D image of an E12.5 *Cx3cr1‐gfp* ^+/−^ mouse pallial slice after agarose‐embedding. The movie shows the 3D image of the E12.5 *Cx3cr1‐gfp* ^+/−^ pallial slice, which was prepared using the agarose‐embedding method, for detecting the GFP signal. This demonstrated that large numbers of intraventricular macrophages associated with the ventricular surface at E12.5 (Figure 3f).

**Video S8.** X‐Y sequential images of an immunostained E12.5 mouse pallial slice after agarose‐embedding. The movie shows dual signals for CD206 (pseudo color green) and DAPI (blue) in the pallial slices of immunostained E12.5 mice treated with agarose‐embedded preparation glass (2 μm pitch) (Figure 3g).
